# Forward genetics identifies cuticle and regulatory genes underlying cellular boundary integrity in *C. elegans*

**DOI:** 10.1093/g3journal/jkag070

**Published:** 2026-04-22

**Authors:** Uroš Radović, Marcus Henricsson, Jan Borén, Marc Pilon

**Affiliations:** Department of Chemistry and Molecular Biology, University of Gothenburg, Gothenburg 405 30, Sweden; Department of Molecular and Clinical Medicine/Wallenberg Laboratory, Institute of Medicine, University of Gothenburg, Gothenburg 405 30, Sweden; Department of Molecular and Clinical Medicine/Wallenberg Laboratory, Institute of Medicine, University of Gothenburg, Gothenburg 405 30, Sweden; Department of Chemistry and Molecular Biology, University of Gothenburg, Gothenburg 405 30, Sweden

**Keywords:** tail tip, membrane, cuticle, permeability, *ptr-18*, collagen, adherens junctions, development, morphology

## Abstract

In *C. elegans*, the epidermis and its overlying extracellular matrix form a primary protective barrier, functioning as the first line of defense against environmental factors. To properly develop those cellular boundaries, a tightly controlled interaction of many molecules and pathways is needed. Mutant alleles of *paqr-2* and *iglr-2* (lipid homeostasis), *dpy-21* (membrane trafficking), *and sma-1* (actin-binding spectrin) result in hermaphrodite tail tip defects suggesting that this simple 4-cell structure can serve as a sensitive model for the identification of pathways responsible for the establishment of cellular boundaries. With this in mind, we performed a small forward genetics screen of ∼800 ethyl methanesulfonate-mutagenized haploid genomes and identified 21 mutants with a Tail End Defects in the hermaphrodite phenotype. Whole genome sequencing of these mutants identified mutations in genes encoding either structural constituents of the cuticle itself (mostly collagen genes) or protein with regulatory functions. By using CRISPR/Cas9 we confirmed 6 novel alleles of *ptr-18, paqr-2, nab-1, ncam-1, vab-9 and efn-4.* We further characterized the loss of function allele *ptr-18(et70)*, which encodes a patch domain-containing (PTCHD) protein homologous to human PTCHD1. *ptr-18(et70)* has a significant effect on growth and development of the worms, while also increasing membrane permeability. Lipidomics analysis revealed no major alterations in membrane lipid composition, implicating cuticle defects as the primary cause of the observed permeability phenotype.

## Introduction

Hypodermal cells in the nematode *C. elegans* form the outer cellular layer of the body and secrete a collagenous extracellular matrix (ECM) that organizes itself into cortical and basal layers separated by struts, forming the worm's cuticle (Cox et al. 1981). During larval development, worms replace their cuticle before each of the 4 molting cycle, a process regulated by the differential expression of many collagen genes (Johnstone and Barry 1996). Defects in cuticle composition and genetic regulation can cause a wide range of body shape phenotypes. Indeed, the genetic basis of morphology in *C. elegans* has been extensively researched by studying mutants with body shape phenotypes such as Long (Lon), Dumpy (Dpy), Small (Sma) and Variable abnormal (Vab), and many of the causative alleles affect the synthesis or trafficking of cuticle components (Brenner 1974). For example, of over 30 characterized Lon and Dpy mutants, 11 affect collagen genes (there are ∼177 collagen genes in the *C. elegans* genome) (Brenner 1974; Cho et al. 2021; Sandhu et al. 2021; Sternberg et al. 2024).

**Fig. 1.**
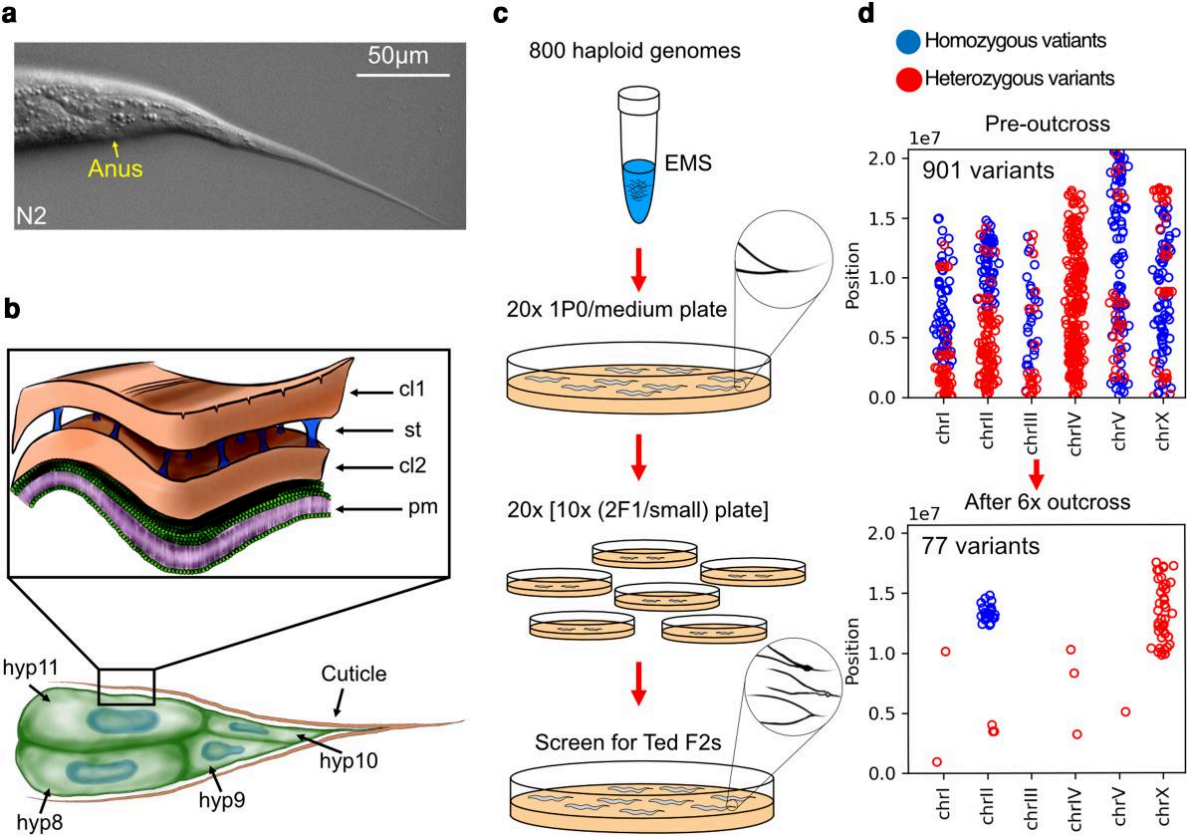
Structure of the tail and overview of the forward genetic screen for Tail End Defects in the hermaphrodite (Ted) mutants. a) Representative tail of a wild-type hermaphrodite showing a long and tapered tail tip. b) The *C. elegans* hermaphrodite tail is comprised of 4 epidermal cells (hyp8 –hyp11) that secrete the cuticle through their plasma membrane (pm). The cuticle has 2 collagen-rich layers (cl1 and cl2), separated by struts (st). c) Outline of the forward genetic screen of 800 EMS-mutagenized haploid genomes screened for Ted mutants in the F2 generation. d) The isolated mutants were outcrossed 6 times and whole genome sequenced to identify clusters of homozygous variants, as shown in this example; the numbers inside the plots represent number of variants found at each step of the outcross.

While gross body shape mutants have been extensively characterized, there is still merit in investigating morphology mutants affecting more subtle or specific structures. For example, mutants that affect the morphology and development of the complex fan-like male tail have provided useful insight into the mechanisms of cell fate decisions and cell fusion (Nguyen et al. 1999; Kiontke et al. 2024). Another structure that merits careful investigation is the long and tapered hermaphrodite tail (Fig. 1a), which is comprised of 4 epidermal cells, hyp-8–hyp-11. Hyp-11 is located dorsally, hyp-8 and 9 ventrally, and hyp-10 creates the extreme end of the tail tip; these cells secrete the tail cuticle, with 2 collagen-rich layers separated by struts (Fig. 1b) (Podbilewicz and White 1994). The hermaphrodite tail is morphologically fragile because it is thin and consists only of hypodermal cells with no underlying muscle, intestine or gonad tissue to provide additional structural robustness. Indeed, some mutations in genes that regulate membrane lipid homeostasis cause tail end defects in hermaphrodites, suggesting that the Tail End Defects in the hermaphrodite (Ted) phenotype may be a useful indicator of membrane homeostasis defects (Svensk et al. 2016a). Specifically, loss of function alleles in the membrane homeostasis regulators *paqr-2* (a ceramidase) and its obligate partner *iglr-2* primarily result in an excess of saturated fatty acids (SFA) within membrane phospholipids that in turn causes intolerance to low temperature or dietary SFA as well as a Ted phenotype (Svensson et al. 2011; Svensk et al. 2013). A previously published screen for mutants that exhibited the same cold intolerance and Ted phenotype as *paqr-2* and *iglr-2* mutants led to the identification of novel mutant alleles of *paqr-2* and *iglr-2* as well as of *dpy-23* (involved in endosome recycling to/from the plasma membrane) and *sma-1* (encodes a spectrin that underlies and structurally supports the plasma membrane) (Svensk et al. 2016a), adding credence to the possibility that the Ted phenotype may be a useful indicator of membrane homeostasis defects.

With the above considerations in mind, we leveraged the Ted phenotype to search for other genetic regulators of cellular boundaries, i.e. cuticle or membrane. We here report the isolation of 21 novel ethyl methanesulfonate (EMS)-induced Ted mutants, the CRISPR/Cas9 verification of 6 of these genes not previously implicated in morphogenesis, and a more detailed study of a novel *ptr-18* allele.

## Materials and methods

### *C. elegans* strains and cultivation

The wild-type *C. elegans* strain Bristol variety N2, *paqr-2(tm4310), ptr-18(ok3532), jcIs* that carries an *ajm-1::GFP* that expresses the AJM-1::GFP adherens junction fusion protein, *glo-1(zu391)* that lacks auto-fluorescent and birefringent gut granules, and *him-5(e1467)* are available at the *C. elegans* Genetics Center (CGC; USA). The PHX10401 *(ptr-18(syb10401))* strain was created by Suny Biotech using CRISPR/Cas9 and carries the mNeonGreen coding sequence, pDD346, a gift from Daniel Dickinson (Addgene plasmid #133311) was inserted immediately upstream of the *ptr-18* stop codon. The inserted sequence is flanked by 5′- GAAAAATCCGTTCGGTCCGCTGAGCGGGCT -3′ and 5′- aataaaaaattacggaaaacaaaaaagTTA -3′. To make the repaired site resistant to re-cutting, a synonymous mutation (AG to TC) was introduced in the PAM site (AGC). All strains used are listed in Supplementary Table 1.

Unless otherwise stated, worm maintenance and experiments were performed at 20 °C, using the *E. coli* strain OP50 as food. The OP50 strain was re-streaked every 6–8 wk on LB plates and maintained at 4 °C. Single colonies were grown overnight at 37 °C in an LB medium with constant shaking then spread on NGM plates to produce an *E. coli* lawn after incubation at room temperature. Seeded plates were stored at 8 °C for up to ∼2 mo.

### Forward genetics screen for Ted mutants

N2 worms were mutagenized for 4 h in a 0.05% solution of ethyl methane sulfonate (EMS) according to a standard protocol (Sulston 1988). After washing, worms were spotted on a NGM plate for 2 h. Among the spotted worms, we singled out 20 L4 hermaphrodites to new plates (progenitor plates) and allowed them to self-fertilize. F1 progeny from each L4 was separated to 10 plates with 2 F1 worms per plate (i.e. 4 mutagenized haploid genomes per plate) for each of the 20 progenitor plates. Finally, we screened for Ted mutants among the F2 generation. In total, ∼800 haploid genomes were screened. Before detailed characterization, the isolated Ted mutants were outcrossed 6 times to wild-type worms by 3 rounds of: mating the Ted mutant with N2 males then mating the resulting heterozygous male progeny with N2 hermaphrodites, then singling out the hermaphrodite progeny and screening for Ted mutants among their offspring.

### Whole genome sequencing

The genomic DNA of the 6 times outcrossed Ted mutants was isolated and sequenced by Eurofins (Constance, Germany) with a mean coverage varying from 36.32X to 81.81X and their genomes assembled using the 98 Mb *Caenorhabditis elegans* reference genome (Genome assembly WBcel235; NCBI RefSeq assembly GCF_000002985.6). Eurofins applied customized filters to filter out false positive variants using GATK's Variant Filtration module (McKenna et al. 2010; DePristo et al. 2011), and the variants detected were annotated based on their gene context using snpEff (Cingolani et al. 2012). For each of the Ted mutants, a hotspot of homozygous variants was identified, and candidate causative variants were selected for further analysis. Of the 21 sequenced mutants, 6 were experimentally confirmed using CRISPR/Cas9.

### CRISPR/Cas9 genome editing

To recreate and confirm the EMS-induced point mutations, we performed CRISPR/Cas9 genome editing as previously described (Dokshin et al. 2018; Ghanta and Mello 2020). The genome editing was done utilizing homology-direct repair (HDR) mechanisms. The protospacer-adjacent motif (PAM) site of the ssDNA oligo template was flanked by 40 bp homology arms. Additional to the candidate point mutation, silent mutations were also added around the Cas9 cutting site to prevent re-cutting after editing. Design and synthesis of the ssDNA and CRISPR RNA (crRNA) was done by using the Alt-R HDR Design Tool from IDT (Integrated DNA Technologies, Inc.; Coralville, IA, USA), including proprietary modifications that improve oligo stability. The ssDNA oligos, crRNA sequences and genotyping primers used for CRISPR/Cas9 are listed in Supplementary Table 1.

The genome editing components were delivered via microinjection into the worm gonads. The injection mix was prepared using 0.5 μL (10 μg/μL) of the Cas9 enzyme (IDT), 5 μL (0.4 μg/μL) tracrRNA (IDT), 2.8 μL (0.4 μg/μL) crRNA (IDT), 2.22 μL (1 μg/μL) of ssDNA (IDT), 40 ng/μL of *pPD118.33* (*Pmyo-2::GFP*), a gift from Andrew Fire (Addgene plasmid #1596), and nuclease-free water to a total volume of 20 μL. Among the F1 generation, worms expressing the *Pmyo-2(GFP)* reporter were isolated and among their progeny we screened for worms with a Ted phenotype. The editing was confirmed by PCR genotyping and successfully edited genes were additionally confirmed by Sanger sequencing (Eurofins).

### Growth assay

For length measurement experiments, bleach-synchronized L1 worms were plated onto NGM plates with OP50 and incubated for 72 h before being mounted and photographed. For experiments performed at 20 °C and 25 °C the worms were incubated for 48 h. The length was measured using ImageJ.

To determine the distribution of stages at 20 °C and 25 °C, 3 replicates of synchronized L1 worms were incubated for 48 h, mounted and photographed. After calculating the percentage of stage distribution for each replicate, we plotted the mean distribution of the replicates. For each growth assay 20 individual worms were measured.

### Protein model

The PTR-18 protein model is based on the structural prediction by AlphaFold (Jumper et al. 2021) and manually drawn using Affinity Designer.

### Gene expression oscillation

The gene expression oscillation of Ted mutants was plotted based on previously published RNAseq data of *C. elegans* throughout larval development (Meeuse et al. 2020).

### Hoechst staining

Bleach-synchronized L1 worms were incubated at 20 °C or 25 °C and Day 1 adults were washed with M9 then incubated for 30 min at room temperature in a 1 μg/mL solution of Hoechst 34580 dissolved in M9. After staining, the worms were washed twice with M9, mounted on agarose pads and imaged using a Zeiss Axioscope microscope. Anterior pharyngeal and posterior tail regions were used for quantification to avoid interference from intestinal gut granule autofluorescence.

### Lipidomics

For each strain, 4 independent replicates of synchronized L4 worms were grown on 9 cm diameter NGM plates; this was done for each of 2 separate experiments. The worms were collected by washing 3 times with M9, pelleted with as much as possible of the supernatant removed and stored at −80 °C until analysis (Lofgren et al. 2016 ). For lipid extraction, the pellet was sonicated for 10 min in methanol:butanol [1:3] and then extracted according to published methods (Jung et al. 2011). Lipid extracts were evaporated and reconstituted in chloroform:methanol [1:2] with 5 mM ammonium acetate. This solution was infused directly (shotgun approach) into a QTRAP 5500 mass spectrometer (Sciex) equipped with a TriVersa NanoMate (Advion Bioscience) as described previously. Phospholipids were measured using precursor ion scanning in negative mode using the fatty acids as fragments (Ekroos et al. 2003; Ejsing et al. 2009). To generate the phospholipid composition (as mol%) the signals from individual phospholipids (area under the m/z peak in the spectra) were divided by the signal from all detected phospholipids of the same class. The data were evaluated using the LipidView software (Sciex).

### Statistics

Error bars show standard deviations unless otherwise stated. The statistical analysis for worm length measurement, male fertility rate, and PTR-18::mNeonGreen intensity was done in GraphPad Prism with a 1-way ANOVA test to identify statistical significance from the N2 control. For the distribution of worm stages, permeability assay, and PTR-18 protein distribution, a 2-way ANOVA test was done. The statistical analysis of the lipidomics data was analyzed with a student *t-*test in Pythons 3.0 with NumPy, comparing the values between N2 and *ptr-18(et76)* for each FA individually. The plotting of the graphs was done with seaborn and matplotlib (Hunter 2007; Harris et al. 2020; Waskom 2021). Every experiment was repeated at least twice, and the statistical analysis shown applies to the presented experimental results.

## Results

### Forward genetic screen for Ted mutants

Twenty-one Ted mutants were identified by screening the F2 progeny from ∼800 EMS-mutagenized haploid genomes (Fig. 1c). After 6 rounds of outcrossing to wild-type worms, genomic DNA was isolated from each mutant and sent for whole genome sequencing. Outcrossing decreases drastically the number of nonspecific mutations and helps in identifying clusters of homozygous variants most likely to contain the Ted-causative mutation. An example of pre and postoutcrossing distribution of heterozygous and homozygous variants is shown in (Fig. 1d).

### The screen reveals mutations in regulatory genes

**Fig. 2.**
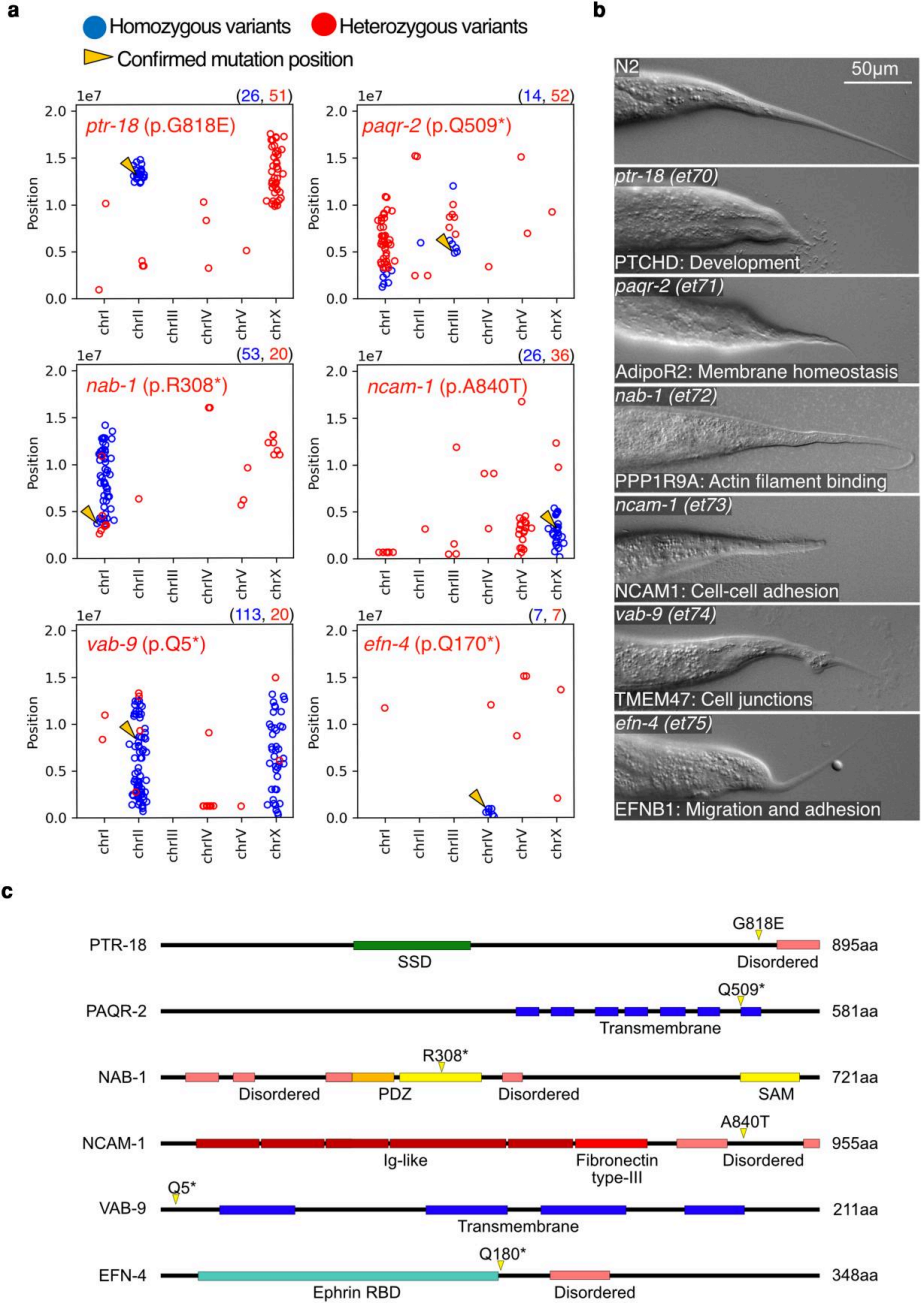
Ted mutants confirmed with CRISPR/Cas9. a) Whole genome sequencing plot showing the position of mutated variant on each chromosome. Red dots represent heterozygous variants; blue dots represent homozygous variants; yellow arrows point to the confirmed mutant alleles positions. The blue and red values above each plot indicate the number of homozygous or heterozygous variants (with frequency ≥0.2) present in the sequenced genomes. b) Tails of Ted mutants isolated in the EMS screen with their genotype (upper left corner), and human ortholog name and function (lower left corner). c) Linear models of the proteins encoded by the 6 CRISPR/Cas9-verified alleles with their functional domains and the mutated amino acid indicated by the yellow arrow.

The 21 Ted mutants showed a variety of distinct tail tip morphology phenotypes, indicating that they likely affect different genes and processes. The whole genome sequencing data was plotted on graphs to show the genomic position of all variants with frequency of 0.2 or more and not present in our wild-type parental strain (which was also sequenced) (Fig. 2a and Supplementary Fig. 1). Assuming recessivity in most cases, we focused on clusters of homozygous GC to AT nucleotide changes (the most common EMS-induced mutation) to identify candidate Ted-causative variants. Among the identified homozygous mutations, missense or nonsense (STOP) mutations in genes previously implicated in developmental processes or cuticle formation were prioritized when selecting for candidates to investigate further. The Ted mutants were then divided into 2 groups. The first group contained 6 Ted mutants with candidate mutations in regulatory proteins and that were confirmed by recreating the point mutations using CRISPR/Cas9 (Fig. 2c and Supplementary Fig. 3). These novel and confirmed alleles are: *ptr-18(et70), paqr-2(et71), nab-1(et72), ncam-1(et73), vab-9(et74) and efn-4(et75)* (Fig. 2a and b). The second group includes Ted mutants that were not CRISPR/Cas9-confirmed because they either contained candidate mutations in cuticular structural proteins or collagen trimmers (13 Ted mutants, including 2 alleles of *dpy-7* and 3 of *sqt-3*) or were mutants for which there are no clear candidate mutation (2 Ted mutants) (Supplementary Figs. 1 and 2).

### Phenotype characterization of Ted mutants

**Fig. 3.**
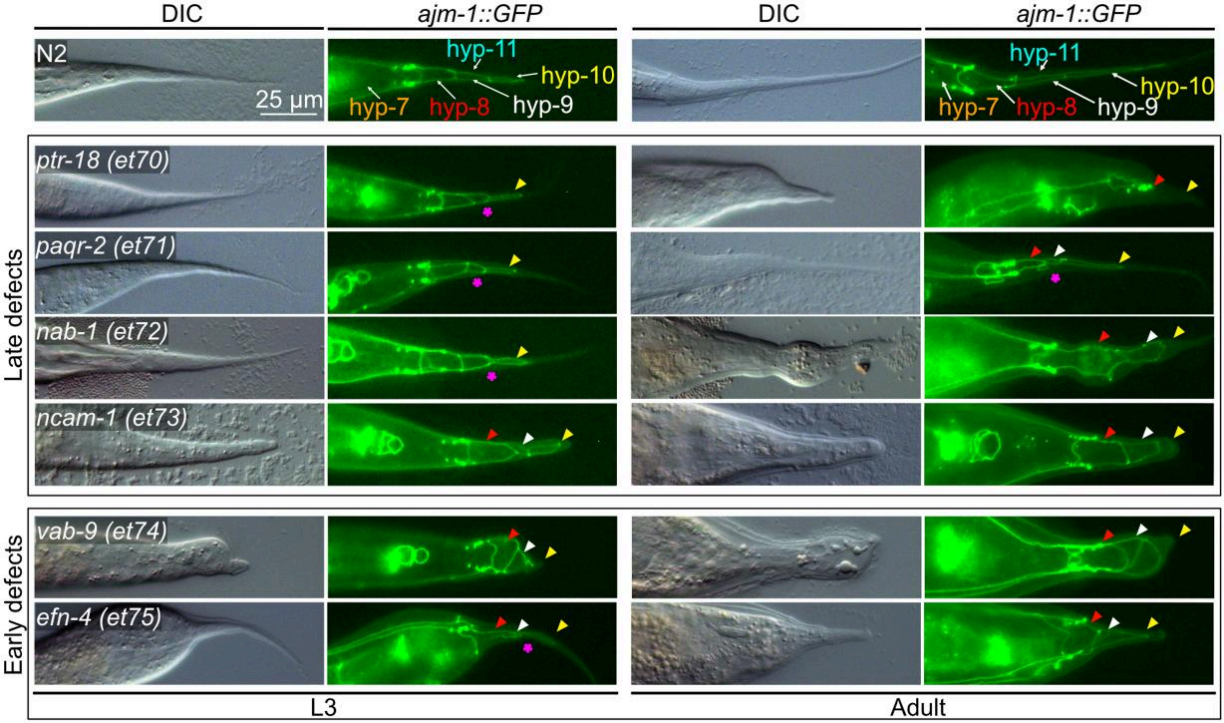
The tail mutants fall into 2 classes: late defects and early defects. An *ajm-1::GFP* reported was crossed into each Ted mutant to visualize the epidermal cells. Each epidermal cell in the tail is indicated by a color-coded arrowhead (hyp-7: orange; hyp-8: red; hyp-9: white; hyp-10: yellow; hyp-11: cyan). Abnormalities of Ted epidermal cells are pointed out with the appropriately colored arrow. Magenta stars point to abnormal constrictions between hyp-9 and hyp-10.

To better understand the cause of the morphological defects in the Ted mutants, we visualized epidermal cells in L3 larvae and adult worms using the adherence junction marker *ajm-1::GFP* (Fig. 3 and Supplementary Fig. 4). All 4 tail epidermal cells were present in all the Ted mutants but showed various degrees of constrictions and deformation throughout development/growth. The Ted mutants fell into 2 groups based on the timing of these morphological defects: a first group with late defects, that initially appear rather normal but in which the Ted phenotype worsens after the L3 stage [including *ptr-18(et70), paqr-2(et71), nab-1(et72), ncam-1(et73)*], and a second group with early defects that show the same phenotype in larval and adult stages [including *vab-9(et74) and efn-4(et75)*]. This classification may reflect distinct roles during post-developmental growth (genes in the first group) vs development per se (genes in the second group).

### Mutation in PTR-18 causes a Ted phenotype

**Fig. 4.**
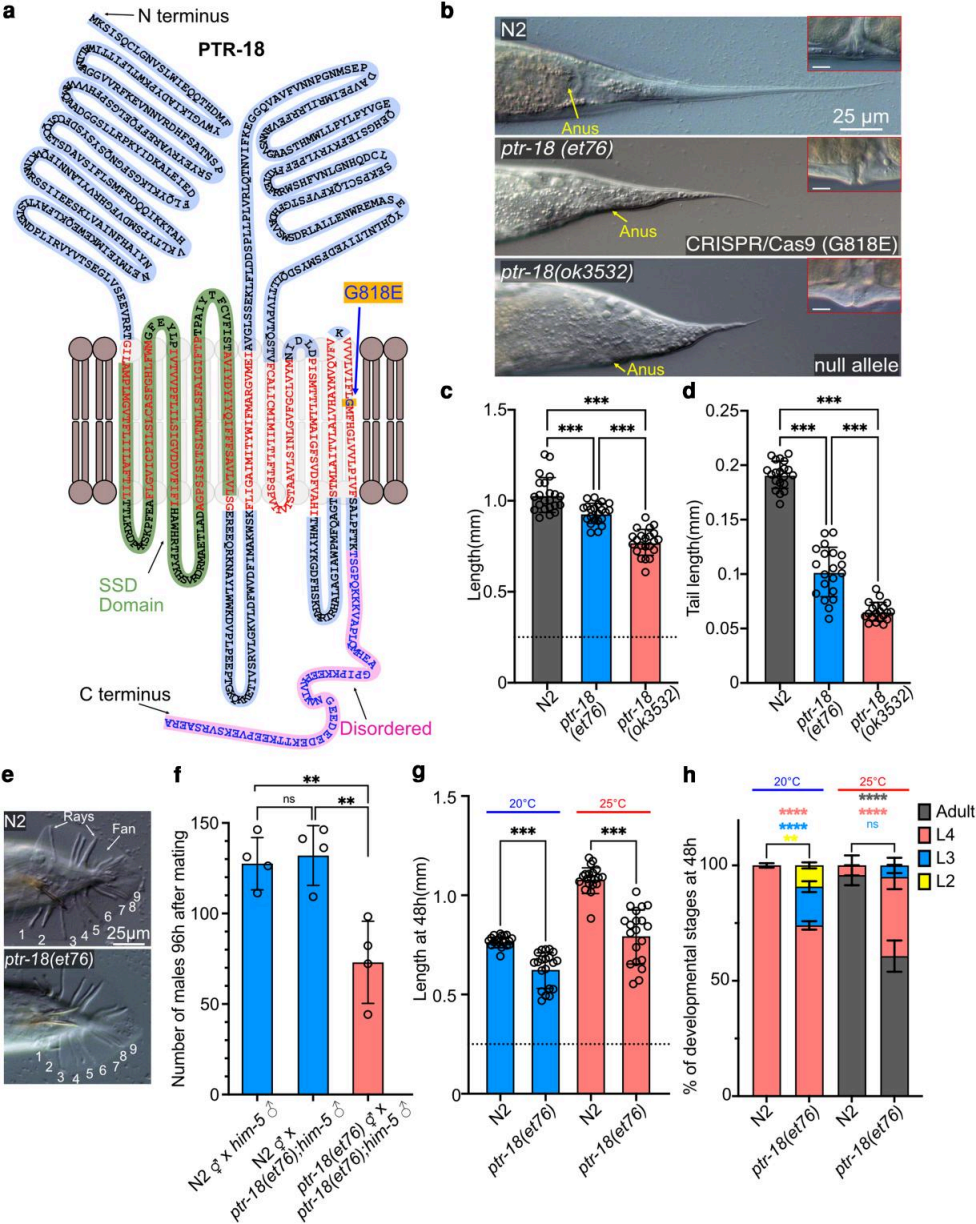
*ptr-18(et76)* is nearly as severe as the null mutant, temperature-sensitive, and does not affect mail tail morphology. a) PTR-18 protein model with the glycine to glutathione amino acid change at position 818 indicated by a blue arrow. The model was drawn manually using Affinity Designer and is based on the structure predicted by AlphaFold (Jumper et al. 2021) b) Comparison between the CRISPR/Cas9-recreated G818E mutation in *ptr-18(et76)* to the *ptr-18(OK3532)* null mutant. Images in red boxes in the upper left corner show the vulva shape of each strain; white line is a 10 μm scale. c) Body length comparison 72 h after synchronization; the dashed line indicates the length of L1 worms at the start of the experiment; *n* = 20 for each strain. d) Tail length of day 1 adults measured 72 h after synchronization; the measurement was taken between the anus and tail tip; *n* = 20 for each strain. e) *ptr-18(et76)* male tail is as in wild-type males. f) Quantification of male fertility by counting the number of male progeny 96 h after crossing 6 *ptr-18(et76)* males with 3 hermaphrodites. Each dot represents the results from a separate cross. g) Body length comparison at 20 °C and 25 °C between wild type and *ptr-18(et76)* 48 h after synchronization; the dashed line indicates the length of L1 worms at the start of the experiment; *n* = 20 for each strain. h) Percentage of larval stages at 20 °C and 25 °C between wild type and *ptr-18(et76)* 48 h after synchronization. A minimum of 50 worms was scored from each of the 3 biological replicates. Error bars show standard error of the mean. ns *P* > 0.05; *P < 0.05, **P < 0.01, ***P < 0.001, ****P < 0.0001 indicate significant difference compared with N2.

Of the 6 Ted mutants that were confirmed using CRISPR/Cas9, perhaps the most interesting is the *ptr-18(et70)* allele, which encodes a patch-related (PTR) protein. PTR proteins are evolutionarily conserved and several reports have suggested that an ancestral function for this protein family may be related to membrane homeostasis or lipid transport and thus contribute to ethanol resistance [*ptr-6* (Choi et al. 2016)], act downstream of *acs-20* [*ptr-8* (Wang et al. 2023)], or regulate oleic acid metabolism under heat stress [*ptr-23* (Venkatesh et al. 2023 )]. The glycine to glutamine substitution at position 818 within the eleventh transmembrane domain (G818E; Fig. 4a) in the novel *ptr-18 (et70*) allele or in the CRISPR/Cas9-recreated allele *ptr-18(et76)* causes a Ted phenotype similar to that of the *ptr-18(ok3532)* null allele (Fig. 4b). However, while both alleles, *ptr-18(et76)* and *ptr-18(ok3532)*, have a similar phenotype, the null mutant is more severe, with a more pronounced protruding vulva (Fig. 4b), shorter body length in day 1 adults, and a shorter tail (Fig. 4c and d); this suggests that the G818E substitution causes a reduction rather than complete loss of function in the protein, i.e. that it is a hypomorph. The function of *ptr-18* is specific to the hermaphrodite tail since the male tail in *ptr-18(et76); him-5* double mutants is unaffected: the shape of the tail and number of rays is as in wild-type (Fig. 4e), and these males were fully fertile (Fig. 4f). Because some *C. elegans* PTR protein family mutations cause heat stress sensitivity (Venkatesh et al. 2023 ), we also characterize the *ptr-18(et76)* at 20 °C and 25 °C. We measured the length of the worms 48 h after L1 synchronization and found that the *ptr-18(et76)* worms had a significantly shorter length at 20 °C compared with N2, and that this growth phenotype defect was accentuated at 25 °C (Fig. 4g). Additionally, while N2 worms uniformly develop within 48 h into L4 larvae at 20 °C, or adults at 25 °C, the *ptr-18(et76)* mutants showed delayed and less uniform development: at 20 °C the mutant worms still had a significant percentage of L2 and L3 larvae, while at 25 °C L4 worms still accounted for ∼28% of the population (Fig. 4h). In conclusion, *ptr-18* is important not only for tail tip morphology but also contributes to developmental rate and robustness, especially at 25 °C.

### Localization of PTR-18 is not affected in the mutant strain

**Fig. 5.**
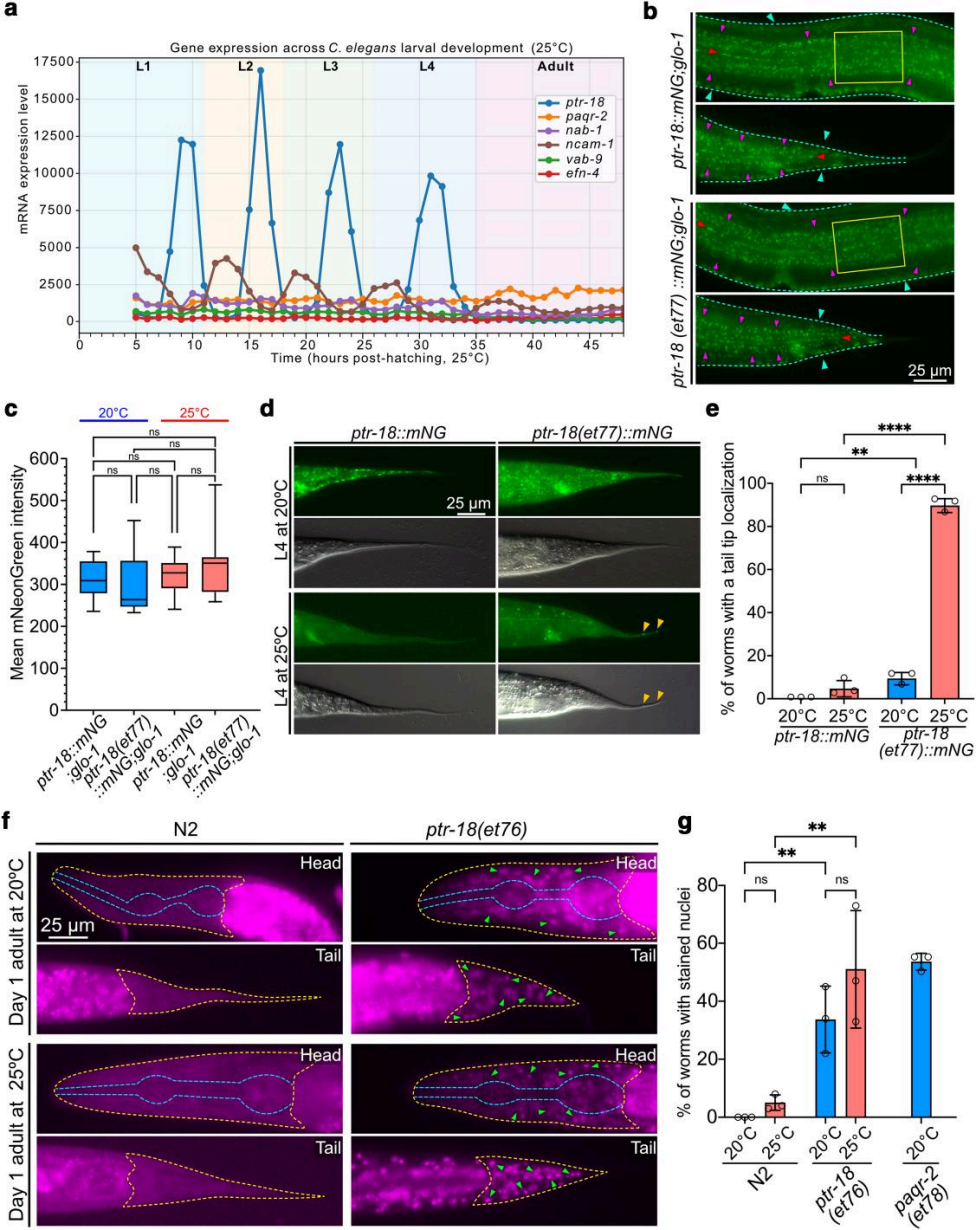
The PTR-18(G818E) mutation impairs the clearance of the PTR-18 protein from the tail epidermal cells during larval development and increases membrane permeability. a) Plot showing the oscillatory gene expression of *ptr-18* and other Ted mutants during larval development (Meeuse et al. 2020). b) PTR-18 protein localization at the L4 larval stage. The endogenous wild-type or mutant PTR-18 coding region was fused at the C-terminal end to a mNeonGreen tag and the modified locus was moved by crossing to a *glo-1* mutant background to remove gut granule fluorescence. Localization of the protein did not differ between the mutant and wild type worms. Dashed cyan line and arrows are pointing to the lateral epidermis distribution of the PTR-18 protein, magenta arrows point to apical distribution of the protein, red arrow points to the localization along the medial body line, and the yellow square points to the area used for measuring the mNeonGreen intensity. c) PTR-18 levels measured through PTR-18::mNeonGreen intensity in L4 worms show no significant difference between wild-type and *et77* (G818E) mutant alleles. To compensate for the oscillatory nature of expression, only worms with arbitrary fluorescence intensity of 230 or more were scored (see also Supplementary Fig. 5c). A minimum of 9 worms was scored for each strain; error bars show standard error of the mean. d) The clearance of PTR-18 is increased at 25 °C in L4 wild type worms, while the mutant strain has a decreased clearance of PTR-18 and is characterized by the formation of protein artifacts in the tail tip (yellow arrows). e) Percentage of worms with PTR-18 artifacts in the tail tip at 20 °C and 25 °C between wild type and *ptr-18* mutant worms. Each dot represents a biological replicate with a minimum of 37 worms per condition. Error bars show standard error of the mean. f) Representative images of wild type and *ptr-18(et76)* worms incubated at 20 °C and 25 °C with Hoechst 34580 in M9 for 30 min show that the *ptr-18(et76)* has permeability defects similar to those in *paqr-2* mutants. Dashed yellow lines outline the head and tail region, dashed blue line marks the pharynx and green arrows point to examples of stained nuclei. g) Quantification of wild type, *ptr-18(et76)* and *paqr-2(et77)* worms with stained nuclei in the head or the tail region at 20 °C and 25 °C. Each dot represents a biological replicate with a minimum of 20 worms per condition. Error bars show standard error of the mean. ns; *P* > 0.05; *P < 0.05, **P < 0.01, ***P < 0.001, ****P < 0.0001 indicate significant difference compared with N2.

To establish the expression level of PTR-18 in different larval stages, we used a published RNA sequencing dataset of gene expression throughout each larval stage (Meeuse et al. 2020). The expression pattern of *ptr-*18 is oscillatory and decreases between each molting event and in adult worms (Fig. 5a). We next used CRISPR/Cas9-engineered strains in which the wild-type or mutant *ptr-18* coding sequence was fused to the mNeonGreen reporter to determine whether the G818E amino acid substitution affects the abundance or localization of the PTR-18 protein. The *ptr-18::*mNeonGreen worms were additionally crossed with a *glo-1* null mutant to remove gut granule autofluorescence. We did not observe any significant changes in the protein localization between N2 and the mutant in L4 larvae: the wild-type and mutant PTR-18 proteins localized in epidermal cells, with signal enrichment along cuticular striations (Fig. 5b). The same localization was found in other developmental stages (Supplementary Fig. 5a and b).To quantify PTR-18 protein levels we incubated L1 worms at 20 °C and 25 °C until they reached the L4 stage, and measured the mNeonGreen intensity in a predetermined region posterior of the vulva in worms that clearly expressed the reporter (PTR-18 expression oscillates and therefore varied significantly even during a same larval stage; Supplementary Fig. 5c). We found no significant difference in the PTR-18::mNeonGreen between the wild-type and G818E variants (Fig. 5c). Even though PTR-18 protein expression levels throughout the larval stages (up to mid-L4) appear normal in the G818E mutant, the mutant protein formed persistent artifacts in the tail tips of late L4 and adult worms while the wild-type protein was uniformly cleared (Fig. 5d). To quantify this observation, we incubated L1 larvae at 20 °C and 25 °C then observed the protein localization once they reached the L4 stage. We found that the G818E substitution caused a temperature-sensitive PTR-18 protein localization phenotype: at 20 °C, the wild-type protein is completely cleared in L4 larvae while the mutant protein shows impaired clearance. Additionally, the formation of protein artifacts in the tail tip significantly increased in the mutant strain from 9% at 20 °C to 90% at 25 °C, and the position of these artifacts correspond to morphological defects visible in DIC images (Fig. 5d and e). Based on the RNAseq data and the oscillatory nature of *ptr-*18 (Meeuse et al. 2020), we know that *ptr-*18 expression declines or ceases completely in adults, leading us to conclude that a consequence of the G818E substitution may be impaired clearance of the protein, leading to the Ted phenotype.

### The membrane of *ptr-18(et76)* mutants is permeable

Mutants in *paqr-2* (Devkota et al. 2021) and in some members of the PTR protein family (Choi et al. 2016) have membrane permeability defects. To test whether this is also the case for *ptr-18* mutants, we performed Hoechst staining on synchronized Day 1 adult worms grown at 20 °C and 25 °C and determined the percentage of worms with stained nuclei in the head or tail region of the worm where auto-fluorescent gut granules do not constitute a confounding factor (Fig. 5f). While there was a small, nonsignificant percentage of N2 worms with permeable membranes at 25 °C due to the increased membrane fluidity at higher temperatures, wild type worms were generally nonpermeable to Hoechst. For *ptr-18(et76)*, the percentage of worms with permeable membrane varied between experiments, with a mean of 33.67% at 20 °C and 51% at 25 °C. As a positive control for the test, we used the CRISPR/Cas9-recreated *paqr-2(et77)* of our *paqr-2(et72)* strain, which showed as expected >50% of worms being permeable to Hoechst at 20 °C (Fig. 5g). We conclude that *ptr-18* is important for establishing impermeable barriers in *C. elegans*, though it is not as crucial for this function as *paqr-2*.

### *paqr-2* and *ptr-18* function in separate pathways

**Fig. 6.**
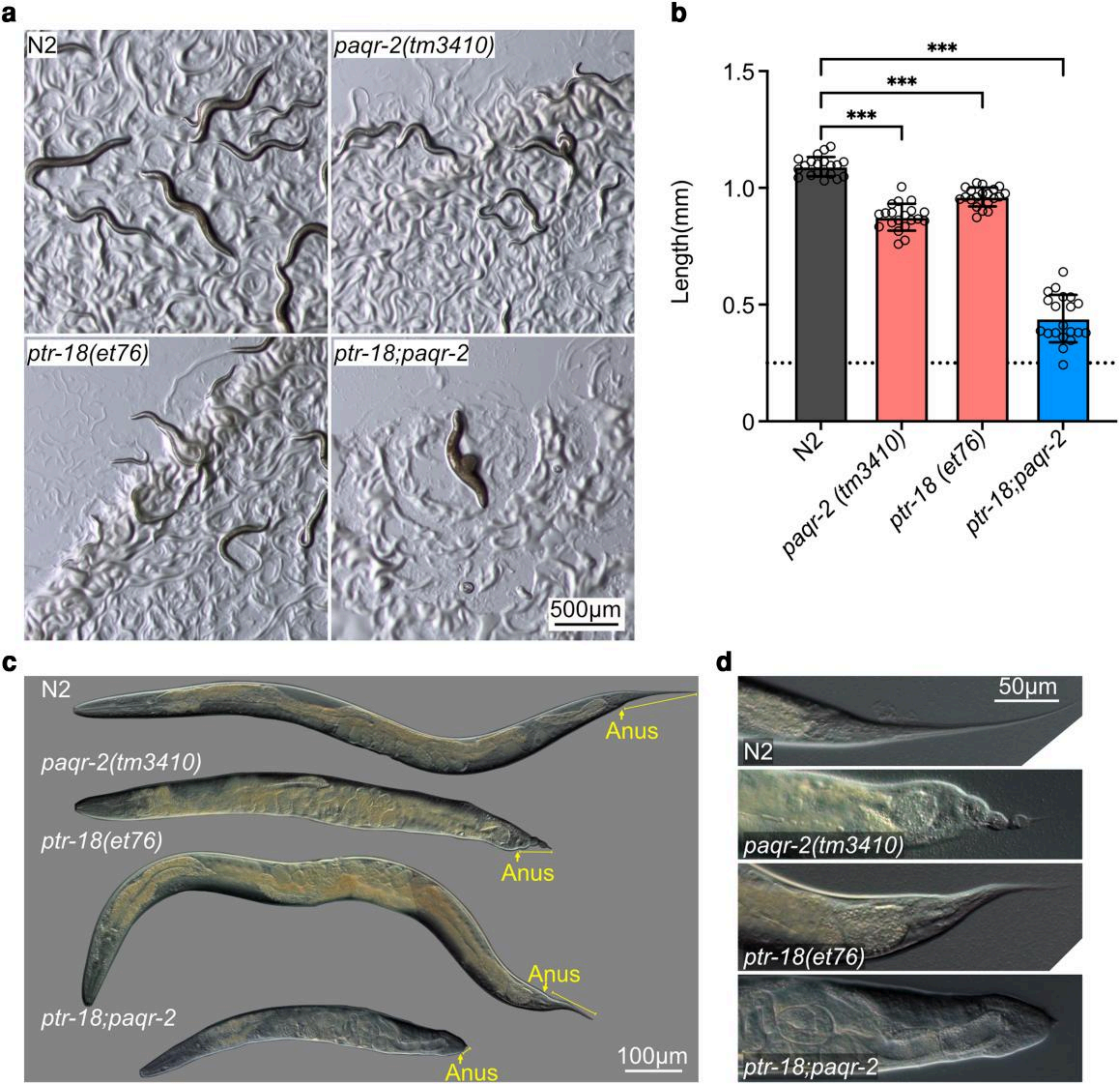
*ptr-18* and *paqr-2* likely act in separate pathways. a) Representative images showing 72 h old plates started with 3 gravid adults for each of the strains. b) Body length measurement 72 h after synchronization; the dashed line indicates the length of L1 worms at the start of the experiment; *n* = 20 for each strain. Error bars show standard error of the mean. *P < 0.05, **P < 0.01, ***P < 0.001 indicate significant difference compared with N2. c) Phenotype comparison of *paqr-2(tm3410)* and *ptr-18(et76)* single mutants, the double mutant and wild type. Yellow arrow points to the anus; yellow line shows the length of the tail. d) Representative images of the tail phenotype for each of the strains.

As mentioned earlier, the membrane homeostasis *paqr-2(tm3410)* mutant also has a Ted phenotype. To explore the possibility that *paqr-2* and *ptr-18* act in the same pathway we created a double mutant. Since both the newly isolated *paqr-2(et78)* allele and the *paqr-2(tm3410)* deletion allele exhibit the same Ted phenotype and slow growth at 15 °C, we used the reference allele *paqr-2(tm3410)* due to its ease of genotyping to generate a *ptr-18(et76); paqr-2(tm3410)* double mutant, and found that it had a significantly worse phenotype than either single mutant in terms of slowed development, decreased reproduction, and production of many L1-arrested progenies (Fig. 6a). We specifically quantified the growth rate of the double mutant and found that it was significantly slower than the single mutants, with a medium length of 0.44 mm after 72 h since the synchronization of L1s, i.e. about half the length of either single mutant (Fig. 6b). The double mutant developed at an inconsistent rate, spanning between the L2 and L4 larval stages after 72 h. The morphology of the adult worms was also significantly deformed, the tail tip posterior to the anus being completely absent (Fig. 6c and d).

### Lipid profile of the *ptr-18(et76)* strain is not significantly affected

**Fig. 7.**
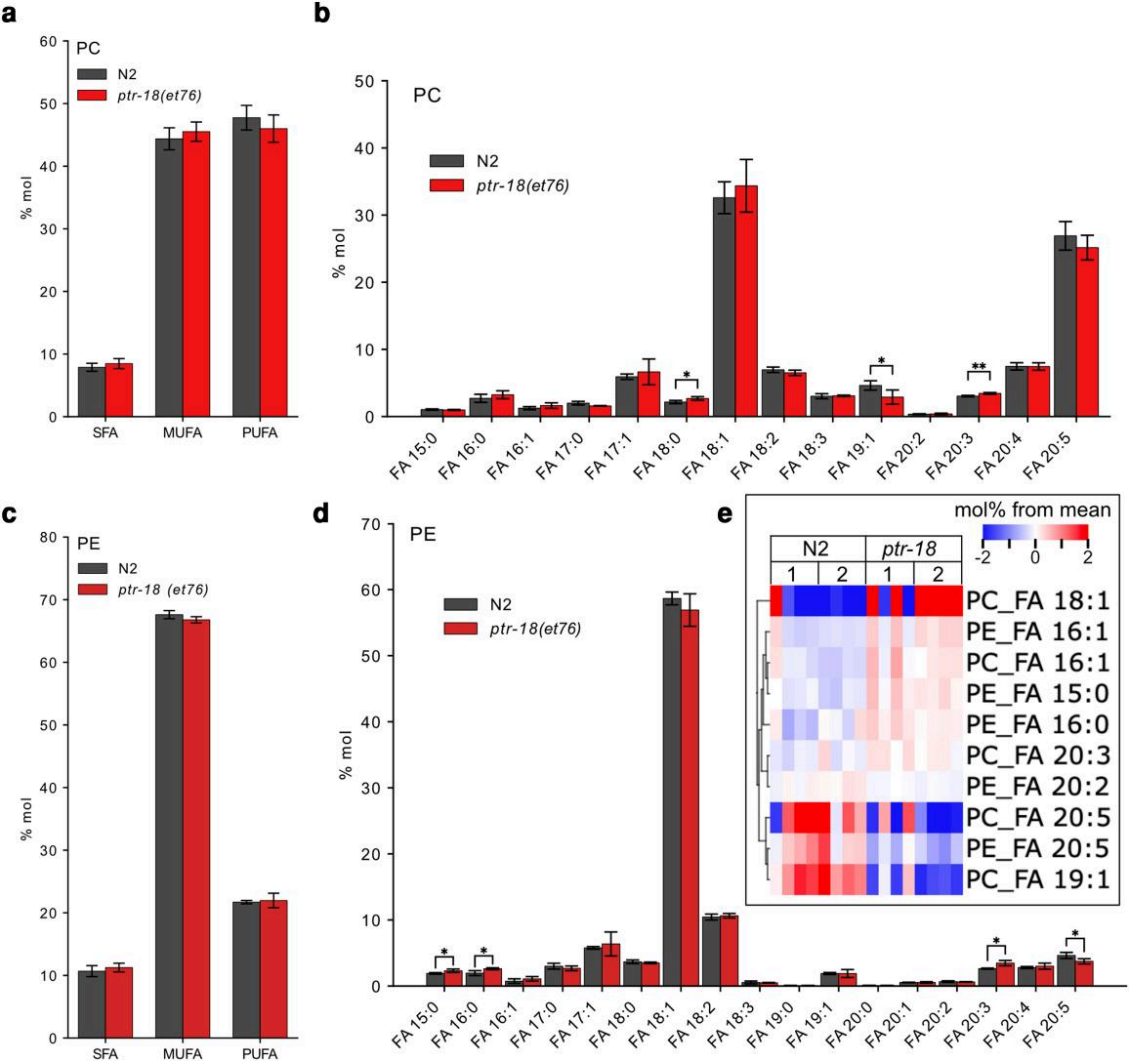
Lipidomics analysis of *ptr-18(et76)* shows no important changes in the lipid composition. a) Levels of SFAs, MUFAs, and PUFAs in PCs between N2 and *ptr-18(et76)* worms are not significantly changed. b) Levels of individual FAs in PCs remain unchanged except 3 FA species (18:0, 19:1 and 20:3). c) Levels of SFAs, MUFAs, and PUFAs in PEs between N2 and *ptr-18(et76)* worms are not significantly changed. d) Levels of individual FAs in PEs remain unchanged except 4 FA species (15:0, 16:0 20:3 and 20:5). *P < 0.05, **P < 0.01, ***P < 0.001 indicate significant difference compared with N2. a–d) shows the mean and standard error of the mean of 4 replicates from the same experiment. e) Heat map showing fatty acids within PCs and PEs that were statistically different (adjusted *P* values of *q* < 0.05 in *t-*tests) between 8 N2 samples and 8 *ptr-18(et76)* samples from 2 experiments, 1 and 2, each with 4 biological replicates per genotype.

The tail tip and permeability defects of the *paqr-2* mutant are caused by an excess of SFA in its membranes (Svensk et al. 2016b; Devkota et al. 2017; Pilon and Ruiz 2023). This is not the case for *ptr-18(et76)* even though it too shows tail tip and permeability defects: lipidomics analyses show that the mutant has nearly normal fatty acid composition in phosphatidylcholines and phosphatidylethanolamines, as well as normal overall levels of SFA, monounsaturated fatty acids (MUFA) and polyunsaturated fatty acids (PUFA). Only the fatty acid 19:1 (decreased in PCs), 15:0 (increased in PEs) and 20:5 (decreased in PEs) showed small but significant and reproducible changes in the *ptr-18(et76)* mutant in 2 separate experiments (Fig. 7a–d shows the results of 1 experiment and Supplementary Fig. 6 shows the other). *t-tests* across both experiments (each comprising 4 biological replicates) suggests that additional fatty acid may be slightly affected in the *ptr-18(et76)* mutant, including 18:1 and 20:5 being slightly decreased in PCs (Fig. 7e). We conclude that *ptr-18(et76)* causes at most only minor changes in the fatty acid composition of the 2 main classes of membrane phospholipids.

## Discussion

With the goal to broaden our understanding of the genetic regulation of cellular boundaries, we performed a forward genetic screen of ∼800 haploid genomes and identified 21 mutants with a Ted phenotype. Six of the novel Ted mutants were confirmed using CRISPR/Cas9 and affect genes with diverse functions: cell adhesion (*nab-1, ncam-1*, *vab-9 and efn-4*) (Wang et al. 1999; Simske et al. 2003; Togashi et al. 2009), membrane homeostasis (*paqr-2*) (Svensson et al. 2011) and trafficking/endocytosis (*ptr-18*) (Chiyoda et al. 2021). It is worth noting that all either encode membrane-bound proteins, or protein that are functionally adjacent to the membrane, supporting the idea that the Ted phenotype is useful to identify regulators of membranes/cellular boundaries.

Several of the novel Ted mutants affect genes implicated in cell adhesion: NAB-1, EFN-4, NCAM-1 and VAB-9. The novel NAB-1 allele affects an adaptor protein (neurabin) linking transmembrane adhesion molecules to the intracellular active zone proteins and the F-actin network during neuronal polarization of axonal synapses formation (Hung et al. 2007; Chia et al. 2012). This is reminiscent of a previously identified allele of SMA-1, an actin-associated spectrin, that also causes the Ted phenotype (Svensk et al. 2016a). While NAB-1 functions in a distinct pathway to SMA-1, both are actin filament-binding protein and may affect tail development through related cytoskeletal mechanisms. EFN-4 is one of the ephrin ligands of VAB-1 (EphR) that regulates cell organization and, when mutated, causes morphological defects also in the head and male tail (Wang et al. 1999). EFN-4 may also function independently of VAB-1 and acts in the epidermis non-cell-autonomously to promote axon guidance and branching (Chin-Sang et al. 2002; Dong et al. 2016; Schwieterman et al. 2016), and we do not know at present if EFN-4 requires VAB-1 for its role in tail tip morphogenesis. NCAM-1, initially characterized as a neural cell adhesion molecule (Vukojevic et al. 2020), may also contribute to the adhesion between the 4 tail epidermal cells in hermaphrodites. As for VAB-9, it is a ubiquitously expressed protein that links the cytoskeleton to adherens junctions and is essential for maintaining F-actin organization and epithelial integrity (Simske et al. 2003). Although our *vab-9(et74)* mutant does not show the same phenotype severity as that of the null mutant (Simske et al. 2003 ), the early stop codon in the protein is clearly enough to cause developmental problems in the tail. Even though some of the functions of NAB-1, EFN-4, NCAM-1 and VAB-9 are already well established, their role in hermaphrodite tail development was previously unknown.

Our screen for Ted mutants identified a novel *paqr-2* allele. The role of PAQR-2 has been extensively studied in the context of membrane fluidity, where it promotes the desaturation, elongation and incorporation of unsaturated fatty acids into the plasma membrane (Devkota et al. 2021; Ruiz et al. 2023). Disruption of membrane integrity and of membrane trafficking that contributes to cuticular component secretion in *paqr-2* mutants could be one of main reasons for the characteristic morphological defects. Another of the confirmed Ted mutants is a *ptr-18* allele, which also encodes a protein that likely contributes to the trafficking of secreted protein (Chiyoda et al. 2021). The PTR-18 protein is homologous to the human PTCHDs (patched domain-containing proteins). As their names imply, PTCHDs have structural similarities to PTCHs (protein patched homolog); however, despite possessing an SSD (sterol-sensing domain) domain like PTCHs, they do not function in the hedgehog pathway. While the underlying molecular mechanisms of PTCHDs are still not fully resolved, proteins like PTCHD1 regulate neural development and synaptic gene expression (Ung et al. 2018 ). Additionally, in line with its role in neural development, PTCHD1 has been associated with intellectual disabilities and autism (Tora et al. 2017). There are 23 PTR proteins in *C. elegans* and they likely have very diverse roles, including lipid metabolism regulation (PTR-8 and PTR-23) and membrane integrity/impermeability (PTR-6) (Choi et al. 2016; Venkatesh et al. 2023; Wang et al. 2023). Even though PTR-18 has an SSD domain, like the mammalian patched proteins, and promotes the clearance of the extracellular hedgehog-related protein GRL-7 (groundhog-like) (Chiyoda et al. 2021), it is important to note that *C. elegans* do not have a hedgehog pathway (Burglin and Kuwabara 2006), leaving the exact role of PTR-18 yet to be discerned. Our research shows that an amino acid substitution in the last transmembrane domain of PTR-18 can have a significant effect on the morphology of the worm. Even though the *ptr-18* mutant has permeability defects similar to that of *paqr-2* mutants, the lipidomics data did not reveal any major changes in lipid composition. Others have suggested that defects in the cuticle could indirectly cause membrane permeability defects (Sandhu et al. 2021; Njume et al. 2022), and this may also be the case for the *ptr-18* mutants. Importantly, while the protein localization during early development was not affected by the mutation, the presence of persistent artifacts in the tail tip points to PTR-18 protein clearance defects between larval stages. Indeed, the PTR-18 oscillatory expression pattern reported here is consistent with an important function during molting and is consistent with previous studies linking PTR-18 with GRL-7, whose localization changes between the matrix and cell during the molting cycle (Serra et al. 2024); lack of PTR-18 leads to an accumulation of GRL-7 in the ECM, causing inappropriate growth (Chiyoda et al. 2021). Although the mechanism of action of the PTR-18 protein remains speculative, it is worth noting that another PTR protein, PTR-23, acts downstream of the collagen DPY-10 in the maintenance of impermeability, likely by downregulating of the desaturase FAT-7 (Venkatesh et al. 2023), suggesting that several PTR proteins may contribute to the regulation of cellular boundaries, from cuticle to phospholipid composition. In this study, we found that the hypomorph *ptr-18(76)* showed only minor changes in phospholipid fatty acid composition indicating that the tail end and cuticle defects in *ptr-18* and *paqr-*2 mutants are possibly caused by different mechanisms.

We did not use CRISPR/Cas9 to confirm 13 of the novel Ted mutants described because they had clear mutations in collagen genes that are structural constituents of the cuticle. Since collagen is the primary component of the cuticle (Cox et al. 1981), it is unsurprising that a majority (13 of 21, i.e. 62%) of the novel Ted mutants carried mutations in genes that encode or regulate collagen, including 2 independent alleles of *dpy-7* and 3 of *sqt-3*. Given that only 800 mutagenized haploid genomes were screened, the sensitivity of Ted phenotype suggests that is especially useful for detecting mutations of the cuticle or ECM.

Screening for mutants with the Ted phenotype can be a powerful approach to identify novel alleles and pathways that regulate development and cell boundary formation/maintenance. The ease with which mutants were identified and the variety of processes that they affect shows that the 4-cell hermaphrodite tail tip is a sensitive structure dependent on many genes for its development (tentatively summarized in Supplementary Fig. 7). The variety of pathways that converge on the same Ted phenotype suggests that tail tip morphology and development depend on multisystem integration. Considering that the worm cuticle is a useful model in research concerning wounding, infection, osmotic stress and development (Chin-Sang et al. 2002), our work suggests that the Ted phenotype will be particularly useful to discover new pathways important for these processes.

## Supplementary Material

jkag070_Supplementary_Data

## Data Availability

The authors affirm that all data necessary for confirming the conclusions of the article are present within the article, figures, and tables. Strains and plasmids are available upon request. Supplemental material available at G3 online.
