# Supplementary material for: Forward genetics identifies cuticle and regulatory genes underlying cellular boundary integrity in C. elegans
Source: G3 (Bethesda). 2026 Apr 22;16(6):jkag070. doi: 10.1093/g3journal/jkag070 (PMC13232528; doi:10.1093/g3journal/jkag070)
Supplement: jkag070_Supplementary_Data [file jkag070_supplementary_data.zip › Figures_S1-S7_G3-2026-406591.pdf]

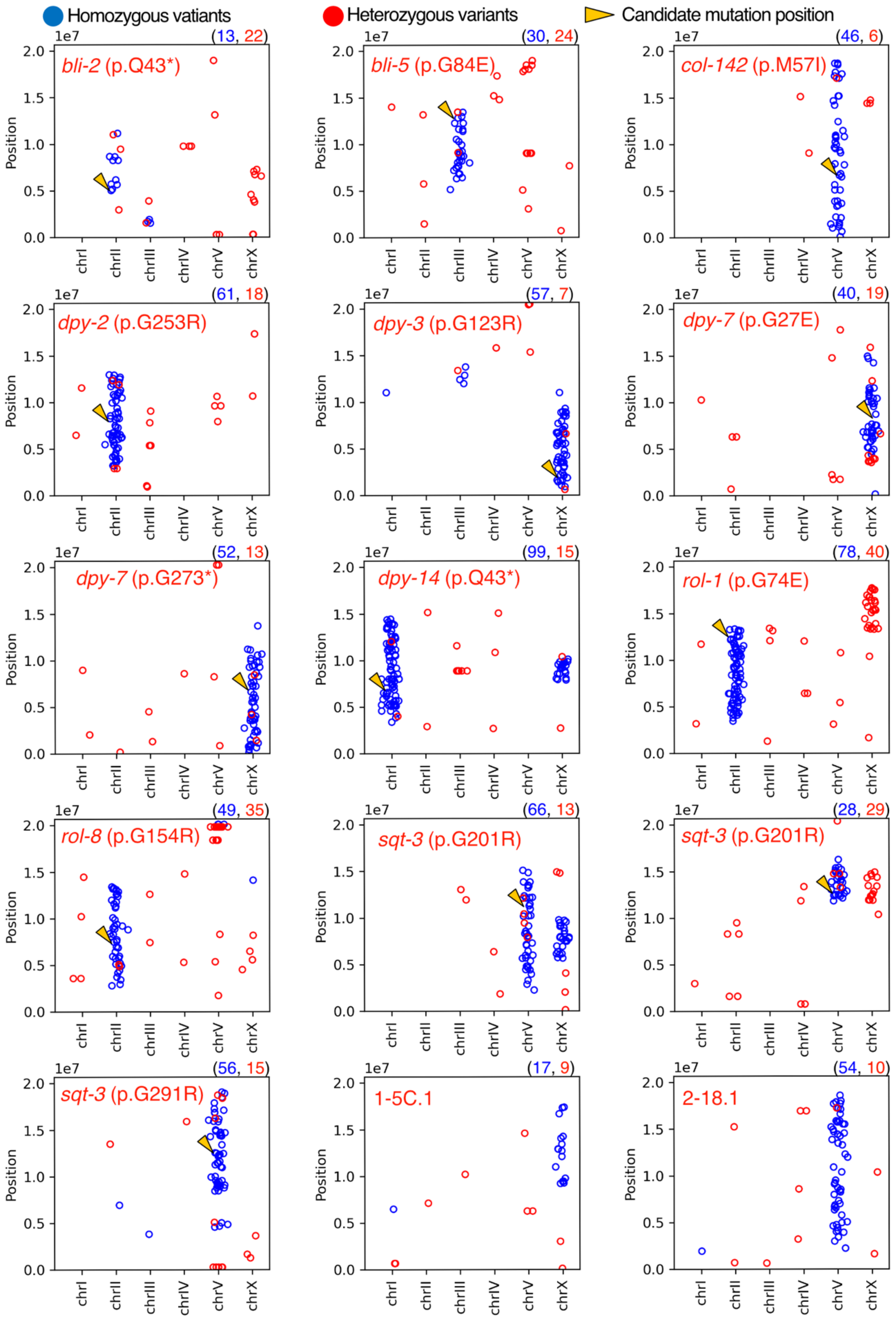

Figure S1. Variant plots for Ted mutants with mutations in cuticular structural proteins or mutants with no clear candidate mutation. Red dots represent heterozygous variants; blue dots represent homozygous variants; yellow arrow is pointing to the confirmed mutant allele position. The blue and red values above each

plot indicate the number of homozygous or heterozygous variants (with frequency ≥ 0.2) present in the sequenced genomes. There were no clear candidate causative mutations for the mutants designated "1-5C1" and "2-18.1".

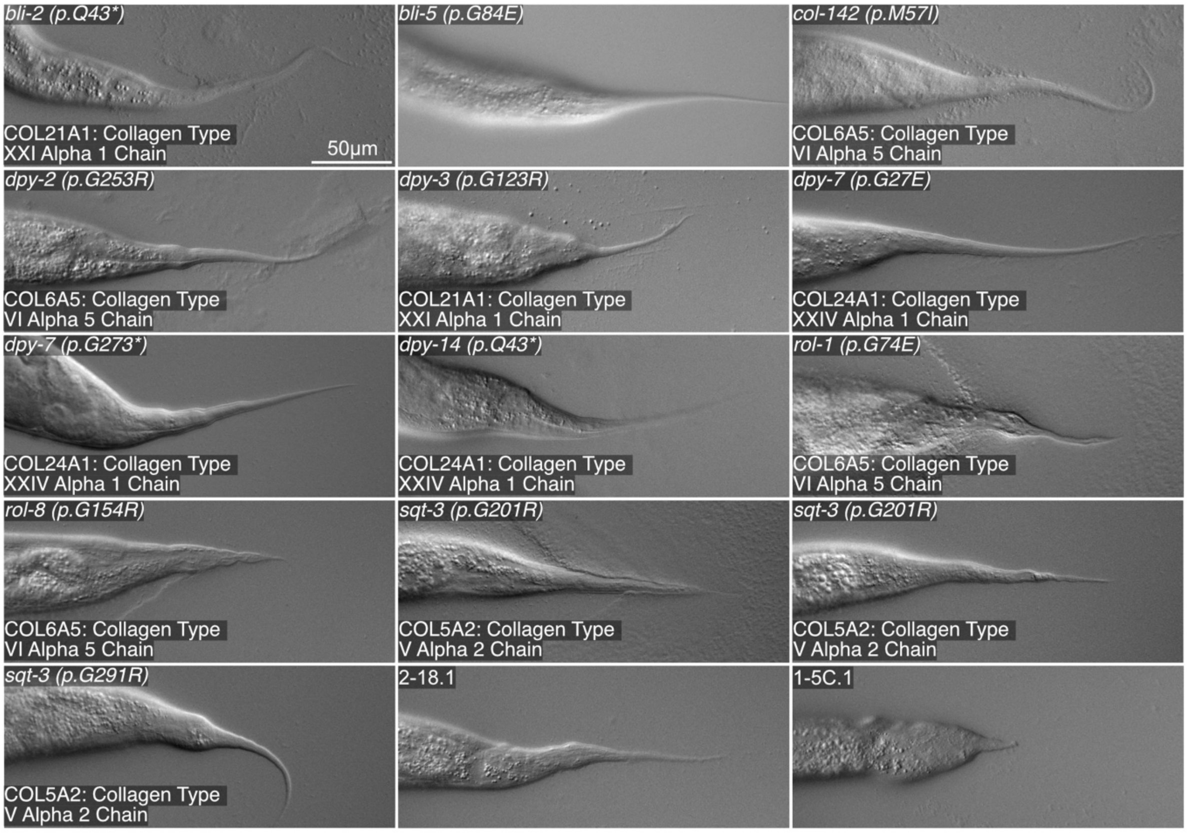

Figure S2. Representative tail images of Ted mutants with mutations in cuticular structural proteins or mutants with no clear candidate mutation. The mutated candidate allele name or designated code name are in the upper left corner and their human ortholog name and function in the lower left corner.

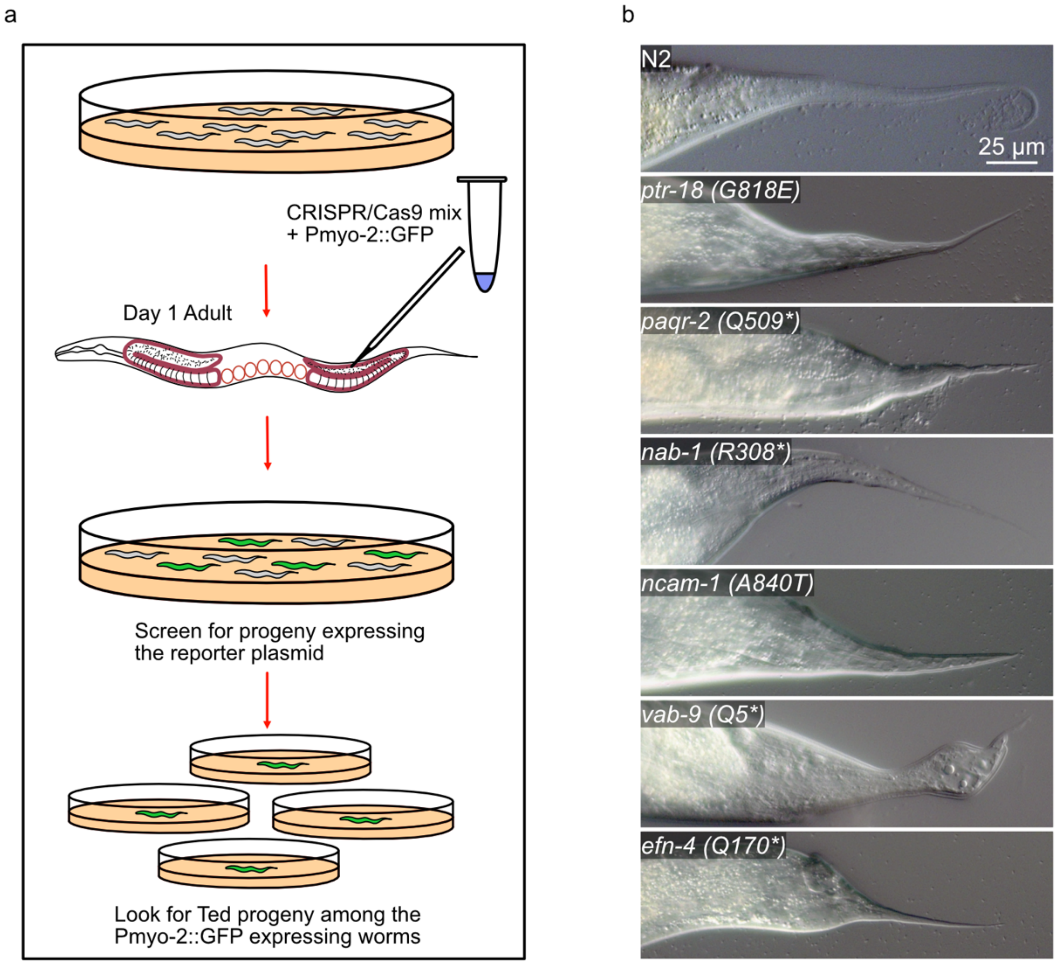

Figure S3. CRISPR/Cas9 confirmation of Ted causing alleles. a) Illustration of the CRISPR/Cas9 microinjection. b) Representative tail images of the CRISPR/Cas9 recreated Ted strains.

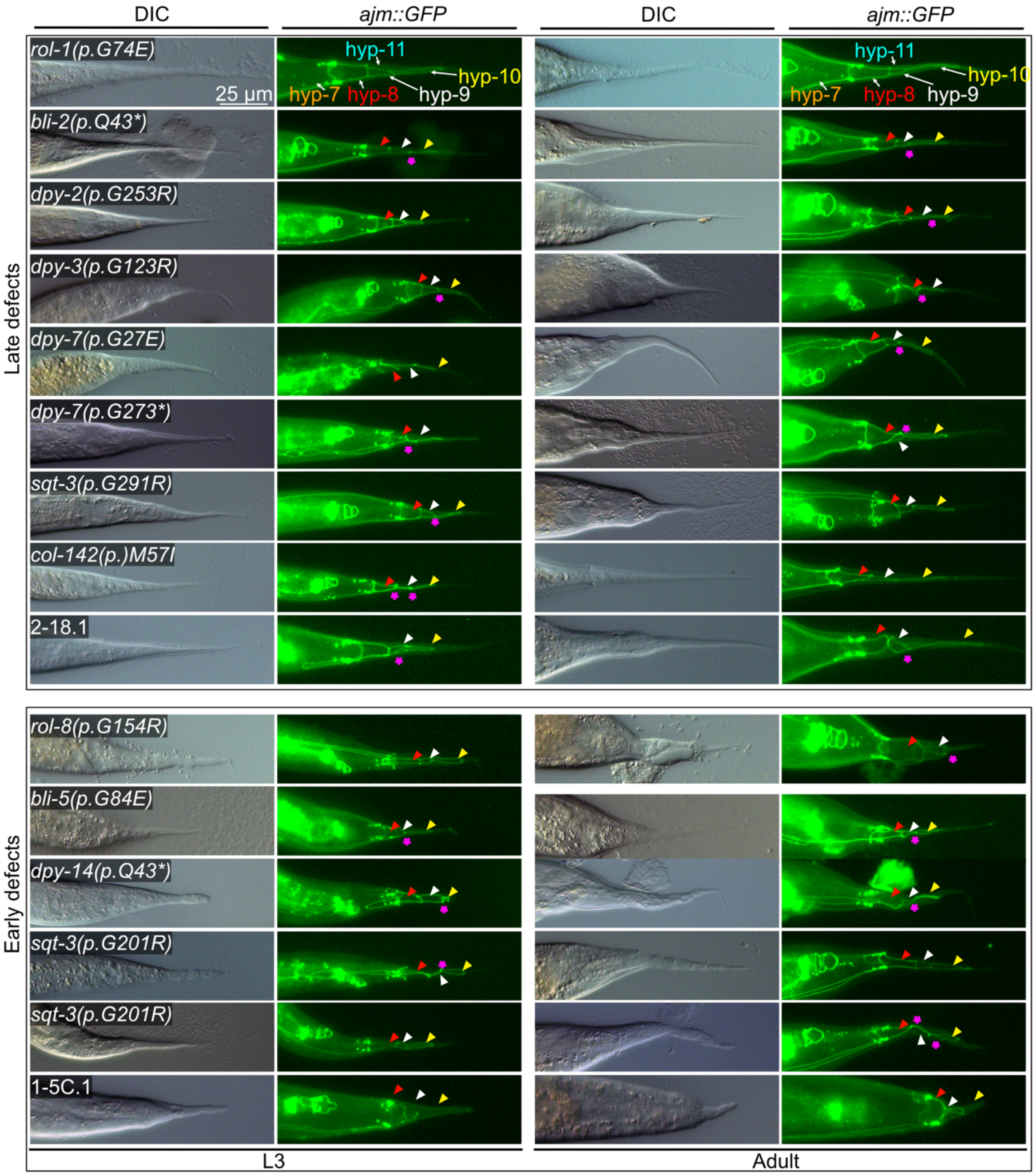

Figure S4. Ted mutants with mutations in cuticular structural proteins fall into two classes: late defects and early defects. An *ajm-1::GFP* reported was crossed into each Ted mutant to visualize the epidermal cells. Each epidermal cell in the tail is indicated by color-coded arrowheads (hyp-7: orange; hyp-8: red; hyp-9: white; hyp-10: yellow; hyp-11: cyan). Abnormalities of Ted epidermal cells are pointed out with the appropriately colored arrow. Magenta stars point to abnormal constrictions between hyp-9 and hyp-10.

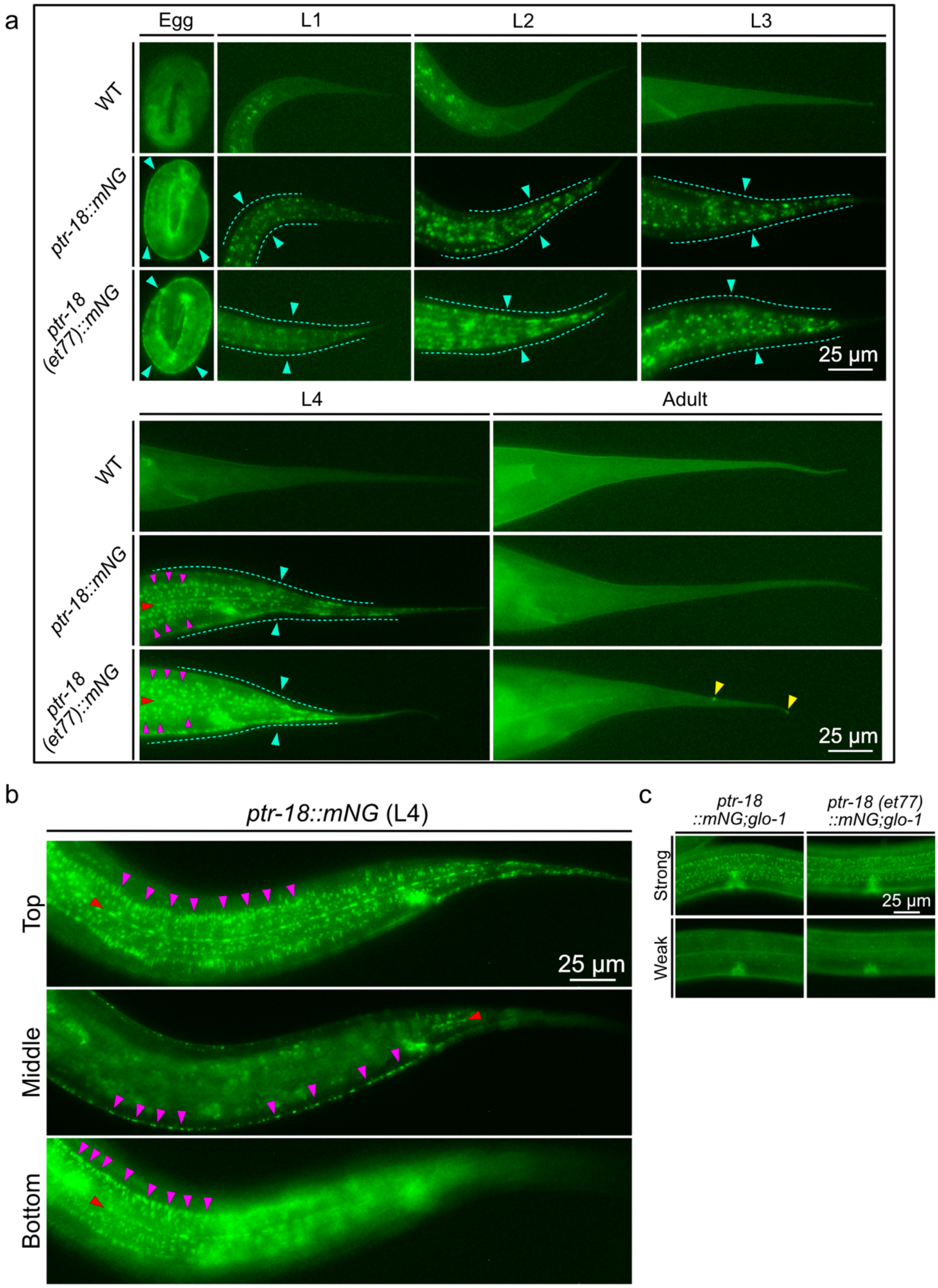

Figure S5. The PTR-18 protein localizes to epidermal cells, along the annuli and seam cells. a) PTR-18 protein localization throughout developmental stages. The PTR-18 protein was labeled with an mNeonGreen tag. Wild type worms were used as a negative control for background autofluorescence. Localization of the protein did not differ between the mutant and wild type worms. Dashed cyan line and arrows are pointing to the distribution of the PTR-18 protein in the epidermis, magenta arrows point

to radial distribution of the protein signal, red arrow points to the localization along the medial body line, yellow arrow point to PTR-18 artefacts. b) Distribution of the protein shown in three different focal planes showing that there is no intestinal localization. Magenta arrows point to PTR-18 signal alignment with the circumferential striations of the cuticle, red arrow points to the localization along the alae. Scale bars in upper and lower panels are identical c) Representative images of L4 WT and mutant worms showing great variability in the protein expression level between individual worms, likely reflecting the oscillatory nature of *ptr-18* expression. mNeonGreen intensity in "weak" worms (e.g. lower panels) was used to set a minimum threshold when selecting worms for protein levels quantification in **Fig. 5c**.

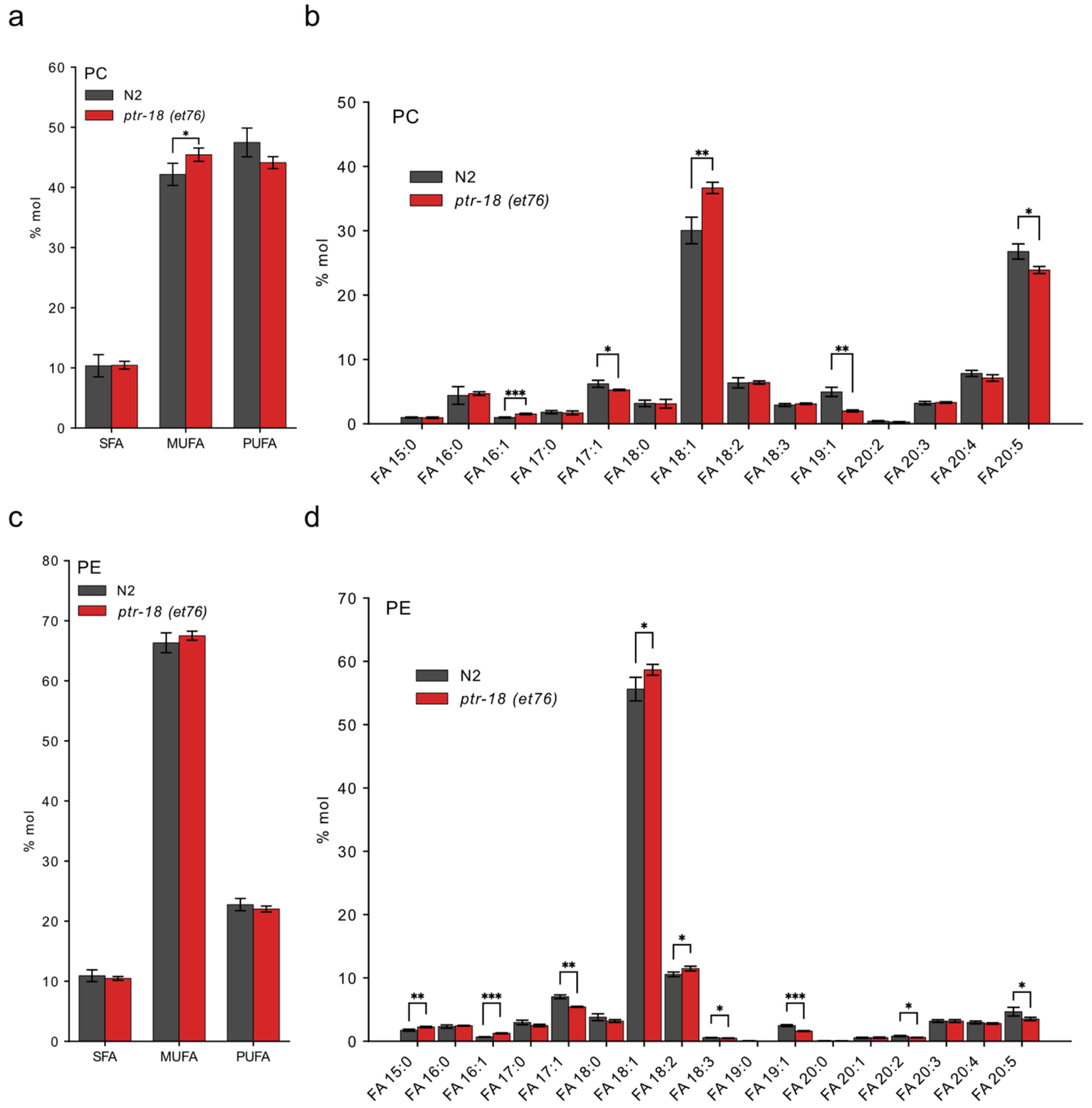

Figure S6. Second lipidomics experiments. a) Levels of SFAs, MUFAs, and PUFAs in PCs between N2 and *ptr-18(et76)* worms were only marginally significant for MUFAs (elevated in mutant). b) Levels of individual FAs in PCs remain mostly unchanged except three FA species 16:1, 17:1, 18:1 and 20:5.c) Levels of SFAs, MUFAs, and PUFAs in PEs between N2 and *ptr-18(et76)* worms are not significantly changed. d) Levels of individual FAs in PEs had significant changes in 15:0, 16:1, 17:1, 18:1, 18:2, 18:3, 19:1, 20:2 and 20:5 FA species. The graphs show the mean and standard error of the mean of four replicates from the same experiment. *p<0.05, **p<0.01, *** p<0.001 indicate significant difference compared to N2.

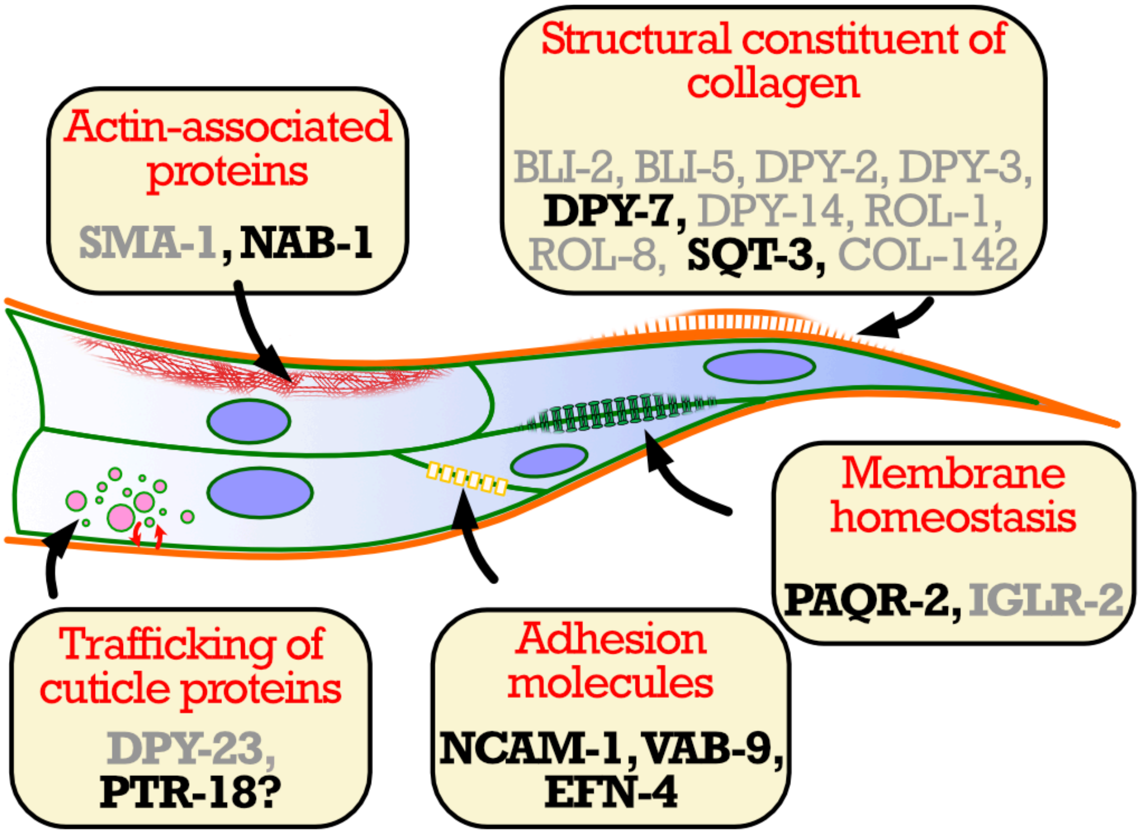

Figure S7. Developmental genetics of the four-cell tail tip. Speculative overview of the developmental genetics of the four-cell tail tip. The morphology and development of the *C. elegans* tail tip is dependent on structural constituents of the cuticle and various regulatory proteins that include membrane homeostasis, adhesion molecules, actin-associated proteins and trafficking of cuticle proteins; PTR-18 is tentatively, hence the question mark, assigned to the latter category.
